# Recent Progress in the Treatment of Fractures

**Published:** 1896-09

**Authors:** 


### RECENT PROGRESS IN THE TREATMENT OF FRACTURES.

In the *Medical News*, July 11th, Dr. A. J. McCosh reviews the most recent methods of treating fractures. He says:

For the treatment of fractures of the lower extremities two new methods, or, rather, modifications of old methods, have been recently proposed in Europe and have already been employed to a considerable extent in the United States. Each of these possesses features of novelty and also of practical utility. Each is worthy of more extensive trial.

The first is the method of "massage and mobilization," which has been systematized and strongly advocated for the past six years by Lucas Championière. He claims that this method is revolutionary and paradoxical, and one absolutely new and contrary to the theory and practice of surgeons. Such terms are, however, extreme, for the plan is not entirely novel, though it has never before been carried into execution in such a systematic and thorough manner as is advocated by this surgeon. According to this method immobilization of the limb is avoided. Massage is begun at once—the sooner the better—and employed daily until the bones have united. The séances as a rule last fifteen to thirty minutes. At first the manipulations must be gentle, and pressure should not be made directly over the ends of the fragments. In the intervals the limb is supported merely by a flannel bandage evenly, but not tightly, applied, or by sand-bags alone. Splints or other immobilizing apparatus are not employed. The claims advanced for this method are: rapid disappearance of pain and swelling, prevention of and more rapid absorption of the edema and infiltration of the soft parts and of the effusion in the joints, preservation of muscular nutrition, and the more rapid formation of a firm callus. As a consequence the fracture is followed by but little stiffness of either muscles, ligaments, or joints, and as soon as the bone is united the function of the limb is fully restored and the patient thus saved weeks or months of pain, stiffness, and disability. In certain cases, however, the originator of this plan grants that his treatment cannot be carried out in all its details. When the mobility of the fragments is so great that there is danger of a permanent deformity, or when the ends are so sharp that manipulation is apt to produce repeated traumatisms of the soft parts, or where the vitality of the skin has been endangered on account of the severity of the injury, the use of massage should be postponed for a week or two and the limb may be even confined for a few days in splints. There can be no question but that in certain cases this plan of treatment possesses advantages over the recognized treatment by complete immobilization. For example, in fractures of the radius or of the fibula, the period of inability will probably be considerably shortened. It is not, however, a safe method to employ in every case of fractured leg. The risk of bony deformity is probably increased and it is very doubtful if either the patient or surgeon will be satisfied with a crooked limb even at the expense of a shorter and an easier convalescence. In many cases this method must prove a dangerous one, and it therefore cannot be recommended for routine practice.

The other method is the so-called ambulatory plan of treatment for fractures of the lower extremity, but especially of the leg. The method is not entirely novel, for at different times it has been used by surgeons in this city, though perhaps not in the systematic manner in which it is now practised in Holland, Germany, and in our own country. Plaster of Paris has always been largely employed in this country and has been especially popular in New York and Boston, in which cities, indeed, its use has been for many years almost universal in the treatment of fractures of the leg. The fracture-box has happily disappeared and in most of our city hospitals is not to be found in the surgical armamentarium. According to the ambulatory plan of treatment the use of the plaster splint is carried to a still greater state of perfection. It is claimed that the patient can get out of bed a few days after the accident, and can walk at the end of the first week, with the assistance of one crutch or a cane. About twelve years ago it became the custom of several surgeons in this city to encourage their patients to get out of bed as soon as possible, and often they would be able to walk on the injured limb at the end of a week or ten days. This plan, however, which only differed in a few details from the method now called the ambulatory, seemed to have fallen into disuse, in part perhaps due to the custom in our hospitals of handing over the treatment of fractured legs to the hospital interns, many of whom have habitually enveloped the limbs in rolls of cotton underneath the plaster. The ambulatory treatment as systematized at the present day consists in the use of a plaster splint snugly applied, without the use of cotton on the leg, but with this addition: that on the sole of the foot underneath the splint is placed a pad of cotton about three-quarters of an inch thick and slightly deeper at the heel. This splint grasps the leg firmly about the bony prominences, especially at the upper part of the leg and head of the tibia. The leg thus hangs suspended to a certain extent in the splint, the greater weight being borne on the bulging part of the leg below the knee, the sole of the foot being separated from the splint by the pad of cotton. The principle is the same as that taken advantage of in the use of an artificial limb, though, of course, the avoidance of pressure at the end of the limb is less perfectly accomplished. In a few cases this splint can be applied immediately before swelling has resulted. Generally, however, this is impossible, and it is then wise to delay its application for four or five days, so that it may not be rendered unserviceable by the shrinkage of the limb. The short period of confinement to bed and the preservation of muscular nutrition are the main advantages of this plan of treatment, and as a result the general condition of the patient remains good and his period of invalidism is much shortened. It is not, however, a method adapted for all cases. In a considerable number the pain is so great that the patients cannot walk; in others, the weight of the splint or timidity prevents locomotion. In some cases the shape of the upper part of the leg prevents proper support; in others the obliquity of the fragments is such that there is considerable risk of overriding and ultimate shortening; and in still others the laceration and contusion of the soft parts is too severe to warrant the early application of the splint. Experience seems to show that the ambulatory splint can be used with distinct advantage in from thirty to forty per cent. of simple fracture of both bones of the leg and in nearly all cases of fracture of the fibula. The same principle may be occcasionally employed with advantage in the treatment of fracture of the knee, though of course the apparatus must be of a different pattern.

One fact must not be forgotten in the trial of new methods—*i. e.*, that there is no class of cases, either in surgery or medicine, where the medical attendant is liable to meet unjust criticism or suits for malpractice as in cases of fractures where a perfect result has not been obtained. Caution, therefore, should be exercised in the employment of methods which have not received the general sanction of surgical authorities.

---

### MARECHAL'S PROCESS OF PRESERVING INSTRUMENTS FROM RUST

A very small amount of alkali is sufficient to keep metal from rusting, so that if steel, iron, nickel, or copper instruments are dipped in five grams alcohol containing one or two grams of either borate, carbonate, bicarbonate, or benzoate of soda, they will not tarnish.—*Gazette Méd. de Liège*, May 14. *Quoted in Journal American Medical Association.*

